# The Importance of User Involvement: A Systematic Review of Involving Older Users in Technology Design

**DOI:** 10.1093/geront/gnz163

**Published:** 2019-11-27

**Authors:** Björn Fischer, Alexander Peine, Britt Östlund

**Affiliations:** 1 Department of Biomedical Engineering and Health Systems, Royal Institute of Technology KTH, Huddinge, Sweden; 2 Copernicus Institute of Sustainable Development, Utrecht University, Utrecht, The Netherlands

**Keywords:** Older technology users, Design practice, Socio-gerontechnology

## Abstract

**Background and Objectives:**

There is a lack of understanding of how older adults’ involvement and participation matters in actual design practice. This systematic literature review investigates existing empirical studies involving older users during the design of technologies and explores the nature and consequences of involving older people.

**Research Design and Methods:**

Our literature review is informed by the guidelines of the PRISMA statement. We examined the included studies by means of thematic content analysis to identify the nature of older users’ involvement and existing evidence on what consequences it has.

**Results:**

In total, 40 empirical studies published in the period 2014–2018 are included in the review. Most empirical studies involve older people from local networks, with underlying stereotypical images and at lower levels of participation. The results reveal three main consequences of involving older users: learning, adjusted design, and an increased sense of participation. Furthermore, we found that user involvement is a structured process whose outcomes are contingent on a range of premises.

**Discussion and Implications:**

Synthesizing the results, we develop the concept of user involvement and present an analytical framework. Our results have implications for researchers and policy makers, since they throw into question the widely held assumption that involving older people inevitably yields beneficial outcomes.

In recent years, the topic of aging in connection with technology has grown in awareness (Pruchno, 2019). It is widely recognized that technologies hold huge potentials to help alleviate the quality of life for older adults. However, barriers to technology adoption by older adults appear to persist (Lee et al., 2019). To help designers develop technologies that more accurately target the needs of older people, “user involvement” is often endorsed as an intervention (Eisma et al., 2004; Peine, Faulkner, Jäger, & Moors, 2015). The main argument put forward for this is the idea that users are experts of their own lives. Hence, involving them can help designers empathize and gain more detailed knowledge about their specific situation: their needs, wishes, and requirements. In turn, the quality of the developed products can be improved (Ives & Olson, 1984), and, in the context of gerontechnology, avoid the occurrence of ageist stereotypes (Frennert & Östlund, 2014). Besides, some scholars have highlighted the active role older users can play as creative sources of innovation (e.g., Essén & Östlund, 2011).

Despite the promises of involving older users in design, a considerable amount of studies raises concerns as to if and how involvement can make a difference in actual design practice. For one, involving users can be fairly demanding, for example, due to risks of misunderstandings between designers and users, difficulties encouraging users, and required time and effort (Wilson, Bekker, Johnson, & Johnson, 1997). Involving representatives of the demographic of older people can be additionally challenging, because older people are an extremely heterogeneous group with highly varied characteristics and needs, who use, modify, and interact with technologies in rather diverse ways (Grates, Heming, Vukoman, Schabsky, & Sorgalla, 2019). Moreover, questions regarding power distributions have been raised regarding participatory approaches in general (Bratteteig & Wagner, 2012) and involving older people specifically (Östlund, Olander, Jonsson, & Frennert, 2015). In situations of such power imbalances, the voice and opinions of older people may be sidelined to make place for more technical concerns.

To achieve acceptable technologies for older adults, hence, an in-depth understanding of how older persons can be involved successfully during technology design is necessary. Does involving older people during technology design matter in practice? And if so, how does it matter? Against this background, the main aim of this article is to provide a review of recent empirical material involving older users regarding how older user involvement matters in actual technology design practice, and synthesize the insights gained with respect to the following sub-questions:

1. What *purposes* are described in the literature for involving older people in the design of a technology?
2. How are older people *selected* for involvement in design practice?
3. What *roles* are assigned to older people when they are involved?
4. At what design *stages* are older people usually involved?
5. At what *levels* of involvement are older people usually involved?
6. What *images* of older people prevail in the literature involving older people?
7. What *consequences* of involving older users are reported in the literature?

This is not the first literature review on user involvement. There are several others (e.g., Bano & Zowghi, 2015; Ives & Olson, 1984; Kujala, 2003; Shah & Robinson, 2007). However, these reviews predominantly focus on the possible benefits and challenges of user involvement, and not on how user involvement matters empirically in *design practice*. Furthermore, most of these reviews focused on management, information systems, or the social sciences, and have thus not considered the *broader literature,* which also includes how user involvement is conducted within the engineering and design fields. Finally, none of the existing literature reviews asked specifically how user involvement matters focusing particularly on *older people*, who are sometimes conceived in terms of stereotypes as a vulnerable and technologically illiterate user group. Our study addresses these gaps and systematically reviews the literature that empirically involved older people, in a broad range of academic disciplines, and with a specific focus on how user involvement matters in those studies.

In our review, we consider that “user involvement” is a broad concept with multiple definitions that are used rather inconsistently in the literature; not seldom synonymous with terms like “user participation” and “user engagement.” Barki and Hartwick (1989) sought to clarify the distinction between user participation and user involvement, indicating that users can be involved without necessarily participating. However, the ambiguous usage of the terms above appears to persist (Bano & Zowghi, 2015). For this review, we defined user involvement in line with Kujala (2003, p.1) as an umbrella term “describing direct contact with users and covering many approaches.” User involvement covers various approaches with different degrees of involvement, the most prominent ones being participatory design (Schuler & Namioka, 1993; Spinuzzi, 2005) and user-centered design (Gould & Lewis, 1985; Norman & Draper, 1986). By “degree of involvement”, we mean the normative degree that users should be involved in the design process based on Arnstein’s (1969) ladder of citizen participation and similar categorizations of the role of users (Mumford, 1979). Such levels usually follow a gradient of the extent to which users exert influence on the overall design process:

- At a low level, users are marginally involved as informants, for example, by filling in questionnaires or testing prototypes in some stages.
- At an intermediate level, users have a more active role, for example, as consultants providing some direct input during the design process but not always.
- At a higher level, users can participate as equal partners, in which they have the possibility to directly influence all design decisions.

Situated at the higher end of the spectrum, “participatory design” is fueled by ideals of empowerment and democracy and has a strong focus on designing for peoples’ purposes (Sanders & Stappers, 2008; Schuler & Namioka, 1993). “User-centered design”, along with other traditions of human-computer interaction design and user experience design, focuses on the improvement of technologies by considering user needs. Here, the expectations for the degree of user involvement are less; users can be involved, for example, in periodic usability tests, by being surveyed or interviewed occasionally during design process, or simply by being observed (Kujala, 2003).

In order to account for the diverse approaches to “user involvement”, we devised our review in a comprehensive manner, as we elaborate below.

## Methods

Our methodology is inspired by the PRISMA statement (Moher et al., 2009). That is, we followed commonly accepted recommendations for conducting systematic literature reviews in line with specified search strategies, eligibility criteria, data extraction guidelines, and analysis methods.

### Search Strategy

We conducted our search by means of five steps: First, we derived major search terms from the research questions. We focused on two specific broad terms (a) “user involvement” and (b) “older adults.” Although we were interested in the involvement of older people specifically during the design of technologies, we refrained from using “technology” as a major search term, because this would have potentially excluded relevant papers that deal with particular technologies, for example, robots or telecare. In a second step, we conducted pilot tests with those broad terms to identify synonymous terms and alternative spellings that are used in current research practice. The identified synonyms are outlined in Supplementary Material, Appendix A. Most notably, we included the various approaches covered by user involvement, specifically participatory design and user-centered design, including their synonyms. Third, we selected electronic databases for our search. We selected *Scopus* and *Web of Science*, because they are particularly relevant for this review, covering a broad range of studies in the fields of social science, aging, and gerontechnology. Fourth, out of our identified terms, we compiled search strings specific for those two databases. To keep our search as broad as possible, we applied our search string to the fields *title, abstract,* and *keywords* in each database. An example search string for Web of Science can be seen in Supplementary Material, Appendix B. We searched for academic articles and conference papers written in English and published in peer-reviewed scientific journals and proceedings within the last 5 years (in the period from 2014 to 2018). In a fifth step, we collected the obtained publications in *EndNote*. The search strategy was discussed and reviewed among all three authors and conducted by the first author.

### Eligibility Criteria

Following this, we discarded duplicate papers and screened the remaining articles for title, abstract, and keywords to filter out studies not relevant for our research topic. In this phase, we excluded the following types of articles:

1. Nonempirical publications, review articles, discussions, presentations, and theoretical contributions.
2. Papers not dealing with older people (aged 50 years or older), that is, studies focusing on caregivers, family members, patients, or other people that were not necessarily old.
3. Articles not explicitly dealing with the development of technologies.
4. Papers in which older people were not involved in the design process. This also concerned papers that did not clearly describe how older people contributed in the process.
5. Predominantly technically focused papers, for example, those dealing with graphics, engineering, and algorithms for computer software.
6. Contributions that solely focused on mental illnesses and cognitive impairments.

For the remaining articles, we obtained full-text versions and assessed them for eligibility based on the abovementioned criteria. In the final review, we only included empirical studies that clearly describe the design process of a technology to which older people at least partly contributed (i.e., at least at the low level). The eligibility criteria were established following ongoing consultations among all three authors. The first author conducted the screening and assessments on both abstracts and full text, whereas the other two authors peer-reviewed the obtained lists of articles.

Systematic reviews sometimes employ quality evaluation methods to assess the strength of evidence of the outcomes reported by the included papers. However, the focus of our systematic review was not on reported study outcomes, but instead on the process of older user involvement. For the purpose of our study, we found that no established evaluation method existed as it was unclear what precisely constitutes the quality of older user involvement. Hence, we opted to not conduct a separate quality evaluation, but instead strived to achieve a high quality of included studies by means of the rigorous eligibility criteria and search strategies outlined above. This choice was also made in previous systematic reviews of involvement processes (e.g., Merkel & Kucharski, 2019).

### Data Extraction and Analysis

For the final set of full-text articles, we developed a data extraction sheet. The gathered data covered information on year, name, country, study design, type of technology and project, the number, age, gender and type of study participants, selection method of participants, role and image of older adults, level and stage at which older people were involved, purpose for which older people were intended to be involved, and the consequences of involving older people. The included articles were analyzed by means of qualitative thematic analyses (Braun & Clarke, 2006): The first author thoroughly read and re-read the included articles and identified initial codes that described significant sentences and phrases concerning the research questions. The obtained codes were peer-reviewed among the authors, compared, and subsequently organized into themes. Specifically, we thoroughly examined the included literature to identify relevant themes with relation to how user involvement matters, in particular the differently reported consequences of user involvement. We also examined the papers with respect to the purposes, stages and levels of involvement, the roles and images of older people, and selection procedures.

## Results

### Study Selection

**Figure 1.**
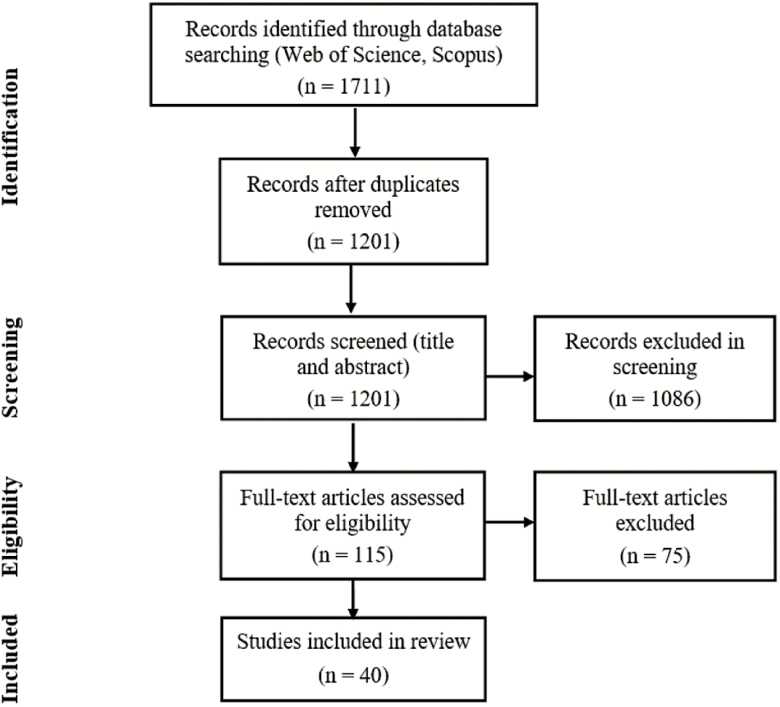
Flow chart of review process.

The initial database search yielded 1711 articles (624 from Web of Science and 1087 from Scopus). The removal of duplicates reduced the number of papers to 1201. Following the screening on abstracts, title, and keywords, in total 115 articles were retained. After we individually assessed those articles on full-text, we included a final amount of 40 studies that met all the eligibility criteria. Figure 1 illustrates a flow chart of the review process.

### Study Characteristics

The 40 included studies deal with the development of a wide variety of different technologies and use a diverse set of methods to involve older people. The technologies that occurred most frequently were mobile applications (30%), robots (17.5%), ambient assisted living technologies (15%), and online platforms (12.5%). The majority of studies focused on the design of a single technology; three studies described the development of more than one technology in a single publication (Frohlich, Lim, & Ahmed, 2016; Joshi & Bratteteig, 2016; Raviselvam, Wood, Hölttä-Otto, Tam, & Nagarajan, 2016). With respect to methods for involving older people, interviews (*n* = 31) were most often employed, followed by prototype evaluations (*n* = 19), focus groups (*n* = 19), prototype tests (including usability testing) (*n* = 17), workshops (*n* = 14), and questionnaires (*n* = 13). Most studies utilized more than one method; only three studies applied just one method (Amos & Lawson, 2017; Le, Reeder, Chung, Thompson, & Demiris, 2014; Wallisch et al., 2018).

The amount of older people involved spanned from 5 to 390 older people. Sixty percent of the studies specified that they involved not more than 20 older people at any given stage. The age spectrum ranged between 50 and 101 years. As related to backgrounds and living environments, seven studies involved care residents, five studies involved older people living at home, and another five studies involved community-dwelling older adults. Additionally, two studies involved both older people living at home and older care residents (Duh, Guna, Pogačnik, & Sodnik, 2016; Haslwanter & Fitzpatrick, 2017). However, 10 studies did not specify the backgrounds of participants, and another 11 studies solely mentioned socio-economic features, such as retirement (*n* = 5), education and retirement (*n* = 2), or age-related diseases (*n* = 3). An overview table of all publications included in the systematic review, specifying detailed information on the extracted data, can be found online in Supplementary Material, Appendix C.

### Purposes and Nature of Older User Involvement

Our thematic analysis revealed that the included studies engaged older people for several purposes that can be summarized by means of three main motivators: (a) Soft motivators, (b) Material motivators, and (c) Normative motivators. Soft motivators referred to purposes of understanding and learning, for example, users’ needs (*n* = 26) and older people’s lives (*n* = 15), as well as obtaining feedback on a particular prototype (*n* = 16). Material motivators denoted intentions to design technology in line with user needs (*n* = 19), achieve a better quality of design (*n* = 11), and increase acceptance (*n* = 10) as well as adoption (*n* = 6). Normative motivators were mostly formulated in the context of achieving empowerment (*n* = 8), and leading to social impact (*n* = 5) like healthier lifestyles and improving life qualities.

Regarding selection procedures, older people were mostly recruited through local networks (*n* = 26), such as affiliated senior centers (Brox, Konstantinidis, & Evertsen, 2017), nursing homes (Compagna & Kohlbacher, 2015), or grassroot organizations (Righi, Sayago, & Blat, 2017). Two studies selected older people online (Mehrotra et al., 2016; Nielsen, Rotger-Griful, Kanstrup, & Laplante-Lévesque, 2018). Several studies (*n* = 11) also reported specific criteria for selecting older people, for example, to only involve active, healthy older adults (Chevalier, Voilmy, Chkeir, & Duchêne, 2018), or to specifically target older persons suffering from a particular deficiency (Hakobyan, Lumsden, & O’Sullivan, 2015). Nine studies did not describe how they selected their participants.

Roughly, the involvement process consisted in three stages, sometimes repeated in iterative cycles: Requirements gathering and design ideation, Technology development and (re-)design, and Prototype test and evaluation. In the studies we reviewed, older people were involved at all of these different stages, but to different extents. Only four studies (10%) included older people at all three stages (Joshi & Bratteteig, 2016; Kopeć et al., 2018; Maaß & Buchmüller, 2018; Righi et al., 2017). Twenty-five studies (62.5%) involved older users at two stages, and 11 studies (27.5%) involved them at one stage. There was a strong tendency to involve users in the first and third stage: On aggregate, there were 32 occurrences of user involvement in the first stage and 34 occurrences in the third stage. At the second stage—the actual design and development process—only seven studies involved older people in some way.

Concerning the roles of older people, there was an overwhelming inclination to involve older people as informants (*n* = 34), testers (*n* = 23), and consultants (*n* = 14). Ten studies employed older people as co-designers, and three studies involved older people as equal partners (Hakobyan et al., 2015; Joshi & Bratteteig, 2016; Kopeć et al., 2018). Regarding the level of involvement based on our adaptation of Arnstein (1969) above, our analysis shows that 70% (*n* = 28) of the studies involved older people at a low level, 22.5% (*n* = 9) at an intermediate level, and only 5% (*n* = 2) at a high level. One exception was the study by Kopeć and colleagues (2018), which described how older people were involved at different levels in different projects, spanning across all three levels.

In this context, our review also foregrounds the governing role of the designers in deciding how older users are involved in practice. For example, the study by Bjørkquist and colleagues (2015) well involved older users to learn about their needs, but the authors problematize that the older people’s influence on different design options was limited, because they were not consulted in any of the main design decisions. Compagna and Kohlbacher (2015) ethnographically investigated the roles of designers and older users during the domestication process of social robots. Their study illustrates how the concerns and suggestions provided by older residents can be consistently ignored by designers, even though they are involved. In some of the studies included in this review, we also found how designers selectively picked information relevant for their technology and feedback to be improved, sometimes deliberately ignoring suggestions by the older people, thereby reducing their influence on the actual design (e.g., Campos, Abade, Silva, & Harrison, 2017; Johnson et al., 2014; Leong & Johnston, 2016; Nielsen et al., 2018; Wu & Munteanu, 2018). In other studies, the designers gave older people more power so they could influence the design to a greater extent (e.g., Eftring & Frennert, 2016; Joshi & Bratteteig, 2016; Righi et al., 2017).

With respect to the images about older people, the majority of the reviewed studies portrayed them in terms of age-related deficiencies, loneliness, or technological illiteracy. This was the case for 90% (*n* = 36) of all studies included in the review. Only four studies employed somewhat positive images of older people, viewing them as technologically skilled (Kopeć et al. 2018), healthy and active (Chevalier et al., 2018; Leong & Johnston, 2016), and as lead users being able to express more needs (however due to age-related decline) (Raviselvam et al., 2016).

### User Involvement of Older People—How Does It Matter?

Our thematic analysis identified three prominent ways that involving older people matters: (1) Learning, (2) Adjusted Design, and (3) Improved sense of participation. We also found consequences of older user involvement for which the literature is ambiguous (4). In the following, we present the findings for each category.

### Learning

Learning is an outcome of older users’ involvement that has been frequently mentioned throughout the studies we reviewed.

One central aspect of learning was that involving older people helped to learn about older people’s needs, even at low levels of involvement. For example, Johnson and colleagues (2014) identified seven different needs for a smart home robot system with the help of older people, including, amongst others, the wish to talk to families and medical caregivers, and getting medication reminders. Similar outcomes were reported by many other studies (e.g., Nielsen et al., 2018; Pollmann, Fronemann, Krüger, & Peissner, 2018). Involving older adults helped the designers to improve their knowledge about older people’s needs. As Leong and Johnston (2016) describe, their idea of developing a companion robot was not in their mind before they engaged older adults. Several other studies also emphasize that the suggestions put forward by older adults led them to contemplate new possibilities for improvements (e.g., Amos & Lawson, 2017; Coelho, Rito, Luz, & Duarte, 2015; Teixeria et al., 2017).

Beyond identifying needs, various papers also note that involving older people helped designers to better learn about older people’s lives. Many studies indicate that involving older people raised their awareness of older people’s daily life practices and activities, such as the importance of social connections (Mehrrotra et al., 2016), common routines for housekeeping (Verhoeven, Cremers, Schoone, & Dijk, 2016), medical practices (Pater, Owens, Farmer, Mynatt, & Fain, 2017), and family visits (Leong & Johnston, 2016). Relatedly, some studies report that the involvement of older people contributed to a better understanding of the lives of older people to the extent that it altered stereotypes. Righi and colleagues (2017) describe how they originally thought of older people as a homogenous group of users, which share a common set of needs that could be targeted by a technology designed for the whole group. The authors extol how involving older people brought to the fore their diverse lived realities, interests, and experiences, shifting their original perception towards seeing older people as a group of heterogeneous, socially active individuals. In another noteworthy study, Kopeć and colleagues (2018) quantitatively assessed the perception of older adults by computer science students before and after the involvement using questionnaires. They found that involving older adults reduced negative stereotypes and improved positive attitudes on nearly all aspects.

Finally, a few studies indicate the relevance of user involvement for mutual learning. These studies commonly employed participatory design and illustrated how user involvement did not only enable designers to learn about older people, but also how it enabled older people to better comprehend design and technology. Lee and colleagues (2017) report how mutual learning occurred as designers learned about the wishes of older adults to maintain control, whereas older adults learned about existing robots, expressing their suggestions more frequently from a design point of view as the design project progressed. Similarly, Joshi and Bratteteig (2016) note how some older individuals developed technological competence over time, and eventually were able to contribute in making technical suggestions.

### Adjusted Design

A second prominent theme in the reviewed literature is the notion that the insights provided by older users lead to adjustments of the design.

One major aspect of this theme is the finding that insights provided by older adults feed into new prototypes. For instance, Le and colleagues (2014) refined their prototype of a visualization of a smart home system in line with the wishes of older adults to provide longitudinal and more detailed information. Similar adjustments are emphasized by several of the studies included in this review (e.g., Brox et al., 2017; Campos et al., 2017; Chevalier et al., 2018; Duh et al., 2016; Harte et al., 2017; Frohlich et al., 2016; Wallisch et al., 2018). These studies commonly highlight one instance of how prototypes have been adapted based on the knowledge obtained through involving older users. A few other studies also indicate multiple iterations during which the insights from older people fed into prototype redesigns (e.g., de Barros, Leitão, & Ribeiroa, 2014; Guo, Zhang, Qian, & Chen, 2016; Kiat & Chen 2015). Here, the authors describe how involving older people throughout multiple iterations aided them to address several issues raised by older adults within their prototypes.

Aside from achieving prototype redesigns, a couple of studies report how older users’ involvement also mattered for the design outcome. One example is the study by Doroudian and colleagues (2018), which explicates how the involvement of older people considerably improved the game design and content, and that their involvement in the design process made the game better tailored to their needs. In a similar fashion, other studies outline how the feedback provided by older users was substantial for the design outcome (Gorkovenko, Tigwell, Norrie, Waite, & Herron, 2017) and significantly altered their design focus (Maaß & Buchmüller, 2018). Furthermore, the study by Kopeć and colleagues (2018) indicates that involving older people at higher levels could improve the overall quality of the technology. The authors observed a 24-hr hackathon in which younger engineers collaborated with older people in different teams. An independent jury then evaluated the final designs from the different teams. The authors find that the two teams that collaborated with older adults at the highest level were the most successful, and their proposed mobile applications won the competition far ahead of the other teams.

### Sense of Participation

The third main way in which we found user involvement to matter was through an increased sense of participation.

For example, Hakobyan and colleagues (2015) conclude that the involvement of older people led to a close relationship among the participants, and that the older people particularly appreciated the feeling of being part of a technologically advanced generation. Maaß and Buchmüller (2018) describe how the older adults valued their involvement in the design project of a digital neighborhood platform, as they felt being treated as an equal partner and expert on their own lives. Eftering and Frennert (2016) note that the older participants appreciated participating in the trials and fondly remembered it as a happy experience. Similarly, Lee and colleagues (2017) remark that the older people enjoyed the workshops as they could socialize and have their voices heard. And Alaoui and colleagues (2014) mention that the participants enjoyed the opportunity to be engaged and contribute with their perspectives.

The experiences reported in the literature can be related to a heightened feeling of ownership among the older participants. As a result of the study by Maaß and Buchmüller (2018) mentioned above, it is explained how the older people became disappointed as they found out that the platform they jointly developed would not be implemented. According to the authors, the older people became so involved in the project that they forgot that the platform was only an exemplary idea. In a similar fashion, Müller and colleagues (2015) report how their older participants valued to see their ideas becoming part of the design. Likewise, Joshi and Bratteteig (2016) describe how they empowered older people to have their voice and say included in different designs, and how many of the older adults appreciated seeing their own ideas take shape over time.

### Ambiguous Findings

Based on our analysis, we found that the literature is inconclusive for whether older user involvement benefits either acceptance or adoption.

First, from the empirical studies we reviewed, the effect of involving older users on technology acceptance remains unclear. On one hand, some studies indicate that involving older users may increase technology acceptance. For instance, Raviselvam and colleagues (2016) evaluated the acceptance of their redesigned tools based on the involvement of older people and found that 90% of older adults and 89% of younger people preferred the redesigned products over existing ones. Several other studies seem to take a positive stance towards the effect of involving older users in the technology’s ultimate acceptance (e.g., Johnson et al., 2014; Maaß & Buchmüller, 2018; Stein, Meurer, Boden, & Wulf, 2017; Wallisch et al., 2018). On the other hand, we also found multiple studies that cast doubt on the positive impact of older adults’ involvement on acceptance. These studies report how older people indicate some positive attitudes to the developed design but provide suggestions for additional features (e.g., Gorkovenko et al., 2017; Guo et al., 2016; Verhoeven et al., 2016), or use it differently than originally intended (Leong & Johnston, 2016; Eftring & Frennert, 2016; Nielsen et al., 2018). Other studies even identified instances of low acceptance. For example, Willard and colleagues (2018) observed that a majority of 73% did not consider the community platform they jointly developed to be of additional value. Also, the prototypes of different mobility aids created together with older adults in a study by Boerema and colleagues (2017) received a low rating on acceptance, both by older people already using similar devices and older persons not using any aids.

Second, we found that the literature is not clear about the impact of older user involvement on technology adoption. In one pertinent study, Haslwanter and Fitzpatrick (2017) historically analyzed how one specific assistive technology project failed to become commercialized even though older people were involved, using mixed qualitative methods and retrospective ethnography. They find that a complex set of factors hampered adoption, including that different user groups were involved in the beginning (older people living at home) than the eventual target group (older people living at residential care facilities), and a neglect of relevant feedback regarding usability and pricing. There are also studies that claim a successful adoption. However, these statements are based on evaluations using indicators from the technology acceptance model (Wu & Munteanu, 2018) and prototype evaluations (Stein et al., 2017), not on an assessment of adoption in the market. Often, authors can only hypothesize that some designs may be adopted in the future (e.g., Kopeć et al., 2018). Most of the studies that empirically involved older users in actual design practice did not evaluate adoption.

In sum, little is known about how older user involvement affects adoption and acceptance. The studies that do consider this topic report ambiguous results, highlighting both positive and negative effects.

## Discussion

### The Concept of User Involvement

Our results enable us to further refine the concept of user involvement in the context of older people. Below, we synthesize our results in order to enhance our understanding of this phenomenon.

The systematic review of empirical studies involving older people fleshed out three principal ways that involvement of older users matters in design practice: The literature provides strong support for that learning, adjusted design and an increased sense of participation can be common consequences of older user involvement. These findings relate to the broader literature. First, the possibility of learning as a desired outcome has been put forward predominantly in the participatory design literature (e.g., Spinuzzi, 2005). Our review adds that learning can be considered as an outcome of user involvement at even low levels of participation, at least in the context of involving older people. It also shows that user involvement can help us to counter stereotypes of older people, which confirms prior suggestions in the aging and technology literature (e.g., Frennert & Östlund, 2014). Second, the review underscores that user involvement can bring about positive feelings among older users related to their sense of participation and ownership. This corroborates the arguments both stipulated in the participatory design (Schuler & Namioka, 1993) and user involvement literature (Tritter & McCallum, 2006), which evoke the position that participation itself matters to those individuals that are affected by design. Third, the review shows that the insights obtained about users can be implemented into the design of the technology and possibly enhance the quality of the design. This finding is consistent with previous knowledge on the potential benefits of user involvement for improved design (Ives & Olson, 1984; Shah & Robinson, 2007), by showing that adjusted design is one potential outcome of older user involvement.

However, while highlighting three different outcomes for which user involvement can matter, our review also revealed that involving older people per se does not necessarily guarantee any specific outcome. Rather, in the literature we reviewed, we found older user involvement to be a structured process whose outcomes appear to depend on a number of premises. For instance, it would be a significant difference whether users are involved to enhance the quality of a given product (material motivator), to achieve that older people have a say in the process (normative motivator), or to provide a basic understanding of the everyday setting in which a new technology needs to operate (soft motivators). Likewise, we found that designers or engineers retain a considerable degree of control about what knowledge from users to include and what to ignore, which role, stage, level, and images prevail, and how participants are selected (e.g., Compagna & Kohlbacher, 2015). These findings concur with earlier suggestions by Sanders and Stappers (2008), who have emphasized the crucial role of designers as facilitators enabling the co-creational capacities of users. They are also compatible with the arguments put forward by Bratteteig and Wagner (2012) and Östlund and colleagues (2015), who stressed that power considerations are crucial during design decisions. Conceptually, hence, older user involvement could best be mapped as a process, which takes different shapes depending on how users are created and enacted in design practice: User involvement can have different outcomes, and the type of outcomes achieved appears to differ with respect to the way designers choose to involve older people.

Moreover, our analysis indicated ambiguous results for the potential outcomes of older user involvement regarding acceptance and adoption of the developed technologies. This finding contradicts results from other reviews on user involvement and success, which appear to find positive links (e.g., Bano & Zowghi, 2015). One possible reason for this contradiction may be that our review specifically focused on the empirical practices of older user involvement in engineering and design practice. It may be that in technology-focused study fields, the economic benefits are not the main interest, as this may be beyond the scope of the respective design projects. However, it is noteworthy that in our review, the empirical design papers are unclear about the impact on acceptance and adoption. Particularly in the context of technologies for older people, the low uptake has been repeatedly problematized (e.g., Lee et al., 2019). Against this background, it appears that more research is needed to develop our understanding of the relationship between older user involvement, acceptance, and adoption.

**Figure 2.**
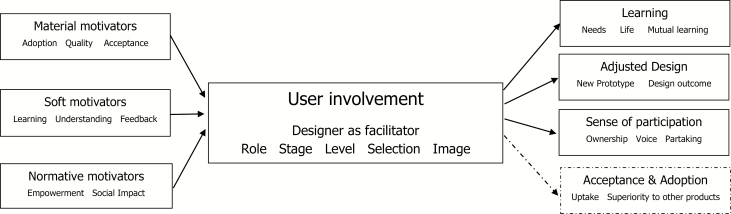
An analytical framework of user involvement of older people.

Below, we summarize the results from this literature review in a stylized model, which outlines older user involvement as a process, including its purposes, nature, and consequences (Figure 2). In dashed lines are the factors that require further research due to ambiguous findings.

### Broader Implications for Research and Policy

Generally, our literature review has shown that older users’ involvement matters, but its outcomes are contingent. This throws into question the widely held assumption that user involvement inevitably yields beneficial outcomes. Rather, it directs our attention to the possibility that the outcomes of user involvement depend on a range of factors, such as its motivators, power aspects, and user images. When we think about user involvement as a process, we need to be more specific about its premises; how, when, and where users should be involved, with which aims or benefits in mind. Reflecting on existing studies involving older people, we find that the literature is not clear about how different premises relate to different design outcomes. Future research could seek to obtain a better understanding of user involvement as a social process and illuminate the intricate dynamics and experiences underlying such design projects.

In this respect, we note that there was a widespread pattern in contemporary design practice to involve older people at low levels, the requirement gathering and prototype testing stages, and with underlying stereotypical images. Furthermore, participants are mostly recruited from local networks. To fully explore the possible consequences of user involvement, future research could engage older people at levels and stages, and with images and roles that differ from the ones that currently prevail, and select them through other networks. Specifically, one may ask: How will user involvement matter if designers engage the concerns and needs of older people even more openly? Interested scholars may want to involve older people at different levels and stages, from different backgrounds and with different roles.

Finally, we found that the connection with acceptability and market uptake is ambitious and generally not well understood. Yet, it is a widespread and persistent assumption that user involvement is a way to ensure user acceptability and market success. In many funding programs, user involvement is promoted as a moral imperative when it comes to technological innovations and aging (Peine et al., 2015). In light of our review, policy makers might need to exercise caution with statements that link user involvement to market uptake or even everyday use. The literature we reviewed rather suggests that the merits of user involvement pertain to the design process itself, by helping us to enable learning, refine designs, and achieve a sense of participation among older users. In how far this translates into a better viability of the designed objects in the everyday lives of older people, or even their acceptability, remains an open question for further empirical inquiry.

### Limitations

There are a few noteworthy shortcomings for this systematic review. First, the studies reviewed only include those published in the last 5 years. Although the included studies range across diverse disciplines and represent knowledge from contemporary design practices, there is the possibility that studies published earlier may have provided relevant insights. Future research may want to include studies within a longer time span. Second, studies not written in English were excluded. This may create a bias in the results of this systematic review, for example, by basing its main findings on reports from Western cultures. Third, studies focusing on older people with cognitive impairments were excluded from this review, which could cause a bias underrepresenting the diverse demographic of older people. Fourth, we have not conducted a separate evidence quality evaluation, as established methods to appreciate the quality of involvement processes were lacking. Our review was designed to improve the knowledge of older user involvement as a process so that such appraisals can be possible in the future.

### Conclusion

Notwithstanding the limitations, this systematic review provided novel insights into the concept of user involvement in the context of older people, highlighting its structured nature depending on a number of premises. Our findings indicate that user involvement does matter for learning, adjusted design, and an increased sense of participation. The review also identified knowledge gaps regarding involving older users, as most studies involve older people from local networks, with underlying stereotypical images and at lower levels of participation. We encourage further research on the relationship with acceptance and adoption, as well as on the actual practices of user involvement.

## Supplementary Material

gnz163_suppl_Supplementary_Material_Appendix_A
