# Supplementary material for: The Importance of User Involvement: A Systematic Review of Involving Older Users in Technology Design
Source: Gerontologist. 2019 Nov 27;60(7):e513–23. doi: 10.1093/geront/gnz163 (PMC7491439; doi:10.1093/geront/gnz163)
Supplement: gnz163_suppl_Supplementary_Material_Appendix_A [file gnz163_suppl_supplementary_material_appendix_a.docx]

**Supplementary Material (to be made available online)**

**Appendix A.** Major search terms including alternatives and synonyms

| 1. User involvement | 1. Older adults |
| --- | --- |
| User involv*  User particpat*  User contribut*  User engag*  Consumer involv*  Consumer participat*  Consumer contribut*  Consumer engag*  Involv* user*  Involv* consumer*  Engag* user*  Engag* consumer*  Participatory design  Participative design  Co-Design  Collaborative design  Inclusive design  Co-production  Co-creation  Participatory technology development  Cooperative design  User-centered  Human-centered  involv* in design  participat* in design  contribut* to design  engag* in design  UX design  user experience design  interaction design | Older adult*  Older person*  Older people  Older user*  Old adult*  Old person*  Old people  Old user*  Aged person*  Aged adult*  Aged people Aged user  Aging person*  Aging adult*  Aging people  Aging user  Senior*  Elderly |

**Appendix B**. Example search string

TS = (("user involv*" OR "user participat*" OR "user contrib*" OR "user engag*" OR "consumer involv*" OR "consumer participat*" OR "consumer contrib*" OR "consumer engag*" OR "involv* user*" OR "engag* user*" OR "engag* consumer*" OR "involv* consumer*" OR "participatory design" OR "participative design" OR "co-design" OR "codesign" OR "collaborative design" OR "inclusive design" OR "co-production" OR "co-creation" OR "participatory technology development" OR "cooperative design" OR "co-operative design" OR "user centered" OR "user centred" OR "user-centered" OR "user-centred" OR "human-centred" OR "human-centered" OR "human centered" OR "human centred" OR "involv* in design" OR "participat* in design" OR "contrib* to design" OR "engag* in design" OR “UX design” OR “user experience design” OR “interaction design”) AND ("older person*" OR "elderly" OR "older adult*" OR "senior*" OR "older people" OR "aged person*" OR "aged people" OR "aged adult*" OR "aging person*" OR "ageing person*" OR "aging people" OR "ageing people" OR "aging adult*" OR "ageing adult*" OR "older user*" OR "ageing user" OR "aging user" OR "aged user" OR “old person*” OR “old people” OR “old adult*” OR “old user*”)) AND DT=(article OR "proceedings paper") AND PY=(2014-2018)

**Appendix C**. Summary of included studies with detailed characteristics

| **Study (year)** | **Purpose of involving older people** | **Technology & Project type** | **Type of older people involved &**  **Selection of participants** | **Study design** | **Role of older users, Involvement level and Stage,**  **Image of older people** | **Reported consequences of older user involvement** |
| --- | --- | --- | --- | --- | --- | --- |
| Alaoui et al. (2014) | Learn about user needs (p.385);  increase acceptance & adoption (p.379) | AAL assistive technologies: Smart TV platform for social interaction & personal life assistant software  Development from scratch | 10 retired persons living alone (1 with arthritis, 4 with computer experience), 65-90 years, 8 women 2 men  Selection through gerontology prevention center, voluntary participation, based on of they were living alone | Semi-structured interviews;  Mockup testing 6 months after interview | Role: informant & tester  Level: low  Stage: early requirement gathering and prototype test  Image: social isolation, frailty, loneliness, age-related decline, lacking technology experience | Answers used to create a set of personas with user attributes and needs, scenarios (p.385f)  Helped to learn about participant’s daily activities and identify areas for improvement (p.383)  Test used to verify design choices, monitor interaction and develop new prototype (p.382, 386)  Participants enjoyed opportunity to provide their perspective (p.387) |
| Amos and Lawson (2017) | Make design inclusive (p.24);    better quality of product (p.21);  meet user needs (p.21) | Portable Fire Extinguisher  Development from scratch | 5 retired persons, older than 60, 3 women 2 men  Selection of residents from a local retirement village | Focus group | Role: consultant & tester  Level: intermediate  Stage: evaluate initial prototype  Image: end user with limitations | Older people highlighted issues that designers were unaware of (p.24)  New concept of technology was developed that incorporated their ideas related to weight, grip and hand strength needed for activation of fire extinguisher (p.25) |
| Bjørkquist et al. (2015) | Identify user needs for future selection of appropriate technology (p.12);  find out how older people can be involved (p.2) | Telecare and telehealth technologies  Improvement of existing technology & finding technology for needs | 42 older seniors (older than 65), 29 women 13 men, and an unspecified number of younger seniors (between 55 and 65),  Older seniors were selected from two community care units where two members of the project management team were employed  Younger seniors were selected from a voluntary community organization. | Focus groups, interviews, a total of 7 interviews were also made with other stakeholders, including relatives, care personal, general practitioners and senior citizen representatives) | Role: informant  Level: low  Stage: early pre-selection phase  Image: patients or service recipients, limited technical knowledge | Needs were identified based on older people’s responses: concern for safety and social relations (p.9ff)  Older people’s influence on choice was limited, designers will make decisions on design and selection (p.15) |
| Boerema et al. (2017) | Identify user values (p.76);  Increase technology acceptance (p.77) | Mobility aids: wheeled walker  Development from scratch | A total of 13 solitary-living, community-dwelling people older than 70 years without cognitive or communicative disabilities: 10 in the first round of interviews, 9 in the second (of which 6 re-joined from the first round; only one male, 9 female)  Selection via a professional homecare organization | Interviews and low-fidelity prototype evaluation | Role: informant  Level: low  Stage: early information gathering phase, and later low-fidelity prototype evaluation  Image: aging individuals who need or may need to use mobility aids in the near future | Values and life attributes were identified (p.78f)  Those served as input for co-creation design sessions where older people were excluded (p.79f)  Acceptance of the three suggested prototypes was low by older people who do not use a mobility aid; and older people who do use one did not see any improvement compared to the one they do use (p.81)  The researchers argue this may have been due to a failure to translate the values into design requirements in the brainstorming session (p.82) |
| Brox et al. (2017) | Improve quality of games to increase satisfaction and acceptance ; to ultimately encourage older people stay physically active (p.1 &10) | Serious exergames to improve older adults’ physical activity  Development from scratch | A total of 16 older persons between 66 and 95 years (beginning: 9, 1 man 8 women, end: 10, 2 men 8 women)  Selection from senior centre that was a partner of the research project; excluded were cognitively impaired, deaf and blind people; included were older people at risk of falling, with physical decline or recent illnesses | Questionnaire, Observation, Videotaping, Group discussion, Structured and semi-structured interviews, prototype testing and evaluation  Regular group meetings every two weeks over a period of 3 years | Role: informant, tester, experimental subject with focus on their frailties and limitations  Level: low  Stage: requirements gathering, user tests  Image: limitations of age regarding speed, movement and visual perception; risk, decline, illness | Many changes occurred based on the insights produced by the various methods, including stronger contrasts and colours, provision of information in the end of the game, slower speed, larger texts and menus, adjusted sounds and movement requirements. (p.8)  Occasionally, based on creative feedback from older people, game story lines where altered (p.7)  The researcher report that involving older people over a long time helped them establish trust (p.11) |
| Campos et al. (2017) | Include older people’s needs and opinions in AAL design (but also of other stakeholders, i.e. managers and carers) (p.552,555, 563) | Ambient assisted living systems  Development from scratch & find useful existing technologies | 9 older people, former teachers, living in a residential home aged at least 70 years. Further involved people were managers and carers, “institutional stakeholders” (they were involved at a higher level and from the onset).  Selection of older people from a private non-profit social-welfare association housing retired teachers | Focus group, virtual rapid prototyping | Role: informant, consultant  Level: low  Stage: evaluation of first prototype  Image: low computer literacy; lacking interest in technology | Based on the input by older people, the different suggested technologies have been adjusted: Guiding lights for bathrooms were removed, automatic calls in case of falls using a bracelet instead of two buttons on door and bed. The idea of locating the residents was augmented with locating them outside the residents’ home, thereby ignoring their comment that they do not need this type of technology for locating them inside the home. Here, the desires of management were prioritized (p.560,564) |
| Chevalier et al. (2018) | Increase acceptance and adoption (p.395)  Increase user satisfaction and usefulness (p.396)  Learn about user needs (p.396) | Software application for motivating older people to be physically active  Development from scratch | 12 retired people (8 women and 4 men) between 62 and 80 years old  Selection based on that participants were still able to be physically active, without serious neurological or sensory decline | Mockup testing and evaluation, collective interview  Feedback discussion | Role: informant  Level: low  Stage: first mockup evaluation (there is a plan to involve the users in later stages too at a potentially higher level, but the research has not advanced there yet)  Image: seniors who are still able to be active, without serious deficiencies; heterogeneous | User feedback has been implemented in new mockup (p.397).  Implemented changes refer to better legibility and information as to where to find elements of the app, and what its purposes were (user-guide video, permanently visible navigation tab bar, changed colours, new welcome screen) (p.397) |
| Coelho et al. (2015) | Learn about user needs (p.114f, 117) | Applications for social connectivity on TV and Tablet  Development from scratch | 31 people older than 60 years during structured interviews (many of which had visual impairments), 20 women 11 men  17 people older than 60 years during focus groups., 14 women 3 men  Selection not specified | 30 Semi-structured interviews  3 Focus groups | Role: informant  Level: low  Stage: early requirement gathering, first prototype test  Image: age-related limitations, loneliness and isolation, lack of technological skills | Replies by older people helped to answer research questions and learn about their preferences (pp.118-123)  Suggestions given by older people were implemented in both prototypes:  -regarding the TV prototype e.g. voice feedback (p.121) and that older people would like sharing photos with their family and friends (p.123) in different sub-groups (p.124)  -regarding the tablet prototype e.g. different ways of organising and navigating the application, such as buttons on both sides for providing help throughout the application regarding which voice commands could be used and being able to see comments and likes (p.125), and additional help in understanding the navigation of the top action bar and shortcuts (p.125)  Comments by older people helped to raise issues the designers had not expected (p.124) for example the ability to share printed photos through the technology (p.126) |
| Compagna and Kohlbacher (2015) | Understand user needs (p.22) | Service robots  Find new use for existing technology | Older people living at a residential care facility (some with light to moderate forms of cognitive impairments but not all)  Care workers and management also formed two user groups  Selection based on contact with nursing home | Observation, open interviews, focus groups, semi-structured interviews, video recording used to develop scenarios  Mediating method: Scenario-based design  Prototype evaluation | Role: informant, tester  Level: low  Stage: early requirement gathering, pilot test  Image: treated like infants, unfamiliar with technology, irrelevant user group, care workers matter more | Inputs from requirement analysis were used to develop scenarios (p.23)  Concerns of and ideas provided by older residents have been consistently ignored by designers (p.24,26).  Reason postulated by researchers: preconceptions about users led to paternalistic stance (p.29f) |
| De Barros et al. (2014) | Evaluate usability of smartphone interface (p.371) | Smartphone application interface designed to promote exercise and prevent falls amongst older adult  Development from scratch | First usability test: 9 people aged between 65 and 92 (7 women, 2 men)  Second usability test: 9 older adults between 68 and 89 (4 women and 5 men)  Third usability test: 7 older adults between 65 and 96  Selection from local day-care centre | Observation of 3 subsequent sessions of usability tests | Role: tester, experimental subjects  Level: low  Stage: testing 3 successive versions of prototype (iterative)  Image: low technological literacy | Insights that surfaced during the tests were considered for the design of the following prototype: insights from the first test were considered for the design of the new interface, like issues with navigating and tapping on text resulted in a new layout with more obvious icons (p.372), and insights from the second test, like older people not perceiving it as making sense to “compete” with someone while dancing, were used in a new language (p.373f), were considered for the third design of the new interface |
| Doroudian et al. (2018) | Learn about needs and interests of older users (p.519)  Receive feedback about design elements of game (p.521)  Empower older adults (p.519) | Digital game to foster social engagement  Development from scratch | 14 cognitively normal adults older than 65 years (10 in first phase, 12 in second, but 8 of those 12 were also present in first phase)  Selection from University’s senior programs to play real-life escape rooms | Observation, Focus group interview  Prototype test & evaluation | Role: informant, tester  Level: low  Stage: Needs assessment, Test of first prototype  Image: not savvy with digital games | The feedback by older people was used as an input for the refinement of the prototype, amongst others: to alter the speed of the game, the navigation, the interface design, the communication, and the controllers (i.e. keyboard and mouse instead of arrow keys). Furthermore, the older adults’ feedback on the concept game resulted in choosing a literary  theme for the game, as well as defining a storyline that connects the puzzles together. The researchers also consider their comments about better real-time instructions. (p.523)  The observations also helped to learn about older users’ needs (p.523)  The authors report that the feedback significantly improved the game design and content (p.523f), and that the older adults’ involvement in the design process could make the game better tailored to their needs (p.516)  However, it is also reported that the older people came up with ideas for the game that were not helpful, impractical or distracted the designers. The researchers attributed this to the lack of familiarity of the older people with technological possibilities (p.523)  In both phases, the older people have suggested a stronger focus on a story line instead of puzzles. The researchers want to wait for the results of a third prototype test before implementing those changes (p.523) |
| Duh et al. (2016) | Learn with which prototypes older adults can better work with (p.92) | Telecare service application  Development from scratch | 45 retired older adults aged between 64 and 91, 32 of whom were nursing home residents and 13 were living independently at home, first field trial: 16 women 2 men, second field trial: 13 women 1 man  Selection and recruitment through the management of a nursing home, based on that they were affected by general age-related changes, but not suffering from special disease | 3 rounds of field trials for prototype testing; Observation | Role: tester, experimental subjects  Level: low  Stage: early prototype testing: low-fidelity and interactive prototypes  Image: requiring medical attention | Observations of users’ interactions were used to improve the service and prepare interactive prototypes (p.92), though it remains unclear how.  The researchers report that often, the older people paid attention to information that was not relevant for the design evaluation, that they experienced difficulties to express their ideas (p.92)  Also, the researchers report that abstract prototypes were difficult for older people to deal with, while using real applications yielded better feedback (p.92) |
| Eftring and Frennert (2016) | Users have no experience with robots (p.274)  Learn about needs and preferences of older people (p.275)  Get feedback from older adults (p.276) | Social assistive robot to prevent falls  Development from scratch | 14 older adults (7 men and 7 women, age 65–86 years) at introduction workshop, 36 older people to complete questionnaire, 14 older persons (3 men and 11 women, age 65–86 years) at qualitative interviews, 18 older adults (age 69–84 years) in 2 design workshops, 7 seniors (2 men and 5 women, aged 72–76 years) in mockup study, 49 older people during laboratory trials, 18 people older than 75 years during home trials  Selection of older adults being interested in robots and/or scared of falling, willingness to remove carpets and move furniture. Selection method not further specified. | Introduction workshop, Questionnaire, Qualitative interviews for gathering requirements, Design workshop  Mockup study, Observation of Laboratory Trials for 2h each, and Feedback from home trials for 3 weeks each | Role: informant, consultant, tester  Level: intermediate  Stage: Needs assessment and user trials; iterative  Image: Older people at risk of falls | Seniors expressed a much more positive view of having a robot in their homes after the workshop and trials (p.276), and they enjoyed partaking in the trials and re-called it as a happy experience (p.280)  A vast array of requirements that surfaced during the various stages of the development process were implemented into the design of the robot, including the ability to clear the floor, search and bring objects, pick up objects from the floor, providing reminders, and displaying and checking physical exercises. It is shown that those changes clearly corresponded with the desires of the older adults (p.277f).  Some requirements could not be fulfilled, like being able to carry shopping bags or provide mobility support (p.278).  In the trials, most participants perceived the robot mostly as entertainment rather than an aid in everyday life. The robot did not increase their perceived feeling of safety at home. Still, they believed that robots will be part of future eldercare. (p.280) |
| Frohlich et al. (2016) | Give older people a voice and role (p.323) | Social media technology  Development from scratch | 18 retired older people between 61 to 80 years old (10 with PC experience, 8 without)  Selection from two distinct networks with ICT drop-in centres | Focus groups, re-design workshops | Role: informant, consultant, tester, co-designer  Level: intermediate  Stage: early prototype evaluation, and re-design  Image: older people have diverse experiences and skills (heterogeneous) | Both groups came up with very creative modifications and extensions of the author’s initial ideas, re-designing and re-purposing a photo-phone into better storing pictures, a TV talking device into having a larger display or being turned into a talking app and a social communication technology into having no keyboard but rather dealing with voice (p.328,329).  The groups responded differently to the same concepts and made different suggestions based on their existing skills and equipment (p.329).  PC users concentrated on technical details and the implementation of features in their discussions. Non-PC users tended to explore the functionality and value of ideas more thoroughly (p.329)  The authors report that the older people desired to design technology such that it would fit with their already existing technology infrastructure (p.329) |
| Gorkovenko et al. (2017) | Generate ideas for design of online application (p.177f)  Learn about older people’s perception of physical and online shopping (p.177f)  Get feedback on usability of prototype (p.179) | Digital device aimed at community-supported online shopping for older people with mobility impairments  Development from scratch | 7 older adults (72-87 years old)  Selection not further specified | Focus group, follow-up feedback session  Rapid prototyping of high-fidelity prototype for evaluation | Role: consultant, co-designer  Level: intermediate  Stage: idea generation, early prototype evaluation  Image: older people with mobility impairments, deteriorating health | To ensure a participant-led focus group, the researchers avoided to steer the participants back to the a-priori defined personas. It has been found that the older people did not relate to the personas and showed little engagement with the technology cards beyond the fingerprint scanner. The main issue that arose because of the unstructured approach was a difference in contribution between outspoken and more reserved participants – with more confident individuals tending to dominate the focus group (p.180f)  The ideas from the focus group served as an inspiration for design, for example the idea to establish security through finger-print verification of purchases, larger fonts, easy English and the participants’ ideas of filtering items by shop, requesting a visit from one of the volunteers, and viewing past purchases (p.179).  In the feedback session, the participants were overall positive towards the implemented ideas (p.181), like the ability to refine results and compare prices between shops and the fingerprint scanner (p.180). They also gave further suggestions, like to have a live video-call function for help and support, or to show special offers at the top of search results (p.180). The participants also wished for a community feature where they could rate visiting volunteers and request the inclusion of new retailers, which the researchers say they will consider in further iterations of the project (p.181). |
| Guo et al. (2016) | Learn about user needs (p.357)  Gather feedback (p.358) | Smart wallet for digital picture exchange and staying connected with children  Development from scratch | 20 older adults from residential care homes, older than 75 years, whose children were living far away  Selected from three residential care homes | Interviews  Prototype evaluation  Prototype test & post-task questionnaire | Role: testers, informants  Level: low  Stage: early requirement gathering, idea selection, early prototype evaluation, high-fidelity prototype test  Image: lonely, lack of cognitive and physical abilities | The outcomes of user requirement gathering were used as an input by designers to brainstorm alternatives. Four developed design alternatives were presented to potential older users, of which one received positive feedback: the smart wallet (p.358-361). This idea was based on the replies by older people that a common way to be involved in their children’s lives was through pictures, and their appreciation of leather products (p.357).  The low-fidelity prototype evaluation showed several crucial usability problems and helped the designers to refine the interface design making it less complicated (p.362), for example to show the most recent picture first (p.363).  The high-fidelity prototype test and evaluation showed that the users were quite satisfied with the product (p.365).  While the designers assumed limited cognitive abilities of older people and therefore did not give them any function beyond looking at the pictures they receive (p.363), the older people wished for a function to respond through pictures themselves, which the designers noted for future design iterations (p. 365) |
| Hakobyan et al. (2015) | Learn about users’ live (p.83)  Learn about user needs and perceptions (p.83)  Empower older adults to directly contribute to design as experts about their lives (p.84)  To better design technology in line with user’s needs (p.79,82)  Increase acceptance (p.79) | Design of a mobile application involving a healthcare diet diary for persons with age-related macular degeneration  Development from scratch | 10 older adults suffering from age-related macular degeneration, at least 50 years old  Selection and recruitment by establishing contact with local community support groups for people with AMD, and attending several of their meetings to establish trust | Focus groups for 4 months, 6 Observational sessions in home  8 Participatory design sessions over 5 months with 4 older people | Role: Informants, co-designers, equal partners  Level: high  Stage: Requirements gathering, design of paper prototypes  Image: provide challenge to healthcare system, patient, experts in living with their condition | The authors report that involving the users was helpful to learn about their needs, experiences and expectations (p.87)  According to the authors, the involvement of participants has improved their opinion of research and their ability to contribute in a meaningful way to research of benefit to them (p.83)  The design sessions resulted in refinements of paper prototypes based on alternative suggestions by participants, such as their suggestion to design a ‘notes page’ to store their ideas and thus support their memory (p.85)  The authors report that a strong bond formed between the participants and the researcher; participants indicated that they could better relate to and feel part of a much younger and technologically- advanced generation (p.88)  Remark: the comments by the authors on the success of the participatory method are held rather vaguely |
| Harte et al. (2017) | Enhance usability and user experience of the app (p.4)  Uncover and mitigate any usability problems as early as possible (p.20)  Elicit feedback (p.4)  Gain understanding of the user (p.20) | Smartphone application to be used as part of a connected health fall risk detection system  Development from scratch | 12 community-dwelling older adults at least 65 years old  Selection based on purposive sample to exclude people with cognitive impairments or hearing and vision frailties  Other types of experts also participated (nurses, occupational therapists, physiotherapists, general practitioners, gerontologists, and engineers) | Interviews, questionnaire, performance test  Prototype evaluation  Scoring tests | Role: experimental subjects, testers, informants  Level: low  Stage: early use case evaluation, late usability test (iterative)  Image: age-related decline of all sorts: vision, physical, cognitive | The older adults found 14 problems, which also had been identified by the expert group (p.9)  The identified problems were addressed by the system developers (p.9) Based on the obtained feedback, a working app prototype was developed (p.10)  Where the problems identified could not be addressed directly due technical constraints, user manuals were created (p.12)  The final usability test showed that out of the originally identified 21 problems, only 3 of those surfaced as problems in the final test. The authors are confident, hence, that they could eliminate 18 problems through involving the users (p.20) |
| Haslwanter & Fitzpatrick (2017) | Learn about user needs (p.759)  Develop technology in alignment with needs of users (p.764) | Assistive technology system  Development from scratch | At show home: 100 older people (55-90 years old) living in their own home  Pilot test : Residents in care facility (60-85 years old)  Selection not further specified  Other types of experts were also included, especially care workers at the assisted living facility | Talking to users in show home, quantitative & qualitative assessment of needs; prototype evaluation  pilot study with pre-and post-interviews, market analysis | Role: informants, testers  Level: low  Stage: early prototype evaluation, requirements gathering, pilot test and evaluation (iterative)  Image: less technical, older users are difficult to get input from | Some suggestions by older people were implemented in technology, like the idea to be able to shop for and order groceries (p.759, 764)  Few of the features, even those that were suggested in the evaluation at the show home and evaluated by the market analysis at the start of the project, were used in the pilot test at the care facility (p.761).  For example, the feature for shopping and ordering groceries was not used, as the residents enjoyed the brief walk to the shop, where they might run into someone they knew (p.761)  Some people rejected it from the beginning, most did not show any initiative in trying the system and learning to use it. Others remarked that it was complicated to use (p.761)  The reasons put forward for this disappointment is that the user groups changed, that it was difficult getting input from users, and that people living at residential care facilities would appreciate other types of features compared to older people living at home (pp.766-769)  It has also been stressed that feedback related to usability and pricing were not given enough consideration in the development phase (p.765)  The development of the system has ended and it is not commercially available any more. However, it has been installed in another facility, and it is working there. It is also still considered to be one of the role models of working AAL systems (p.762) |
| Johnson et al. (2014) | Ensure user acceptance (p.195)  Successful adoption of the system (p.196)  Learn about user needs (p.197)  Design system in line with user needs (p.201) | Socially-assistive robot as part of smart home  Development from scratch and improve existing technology | 17 older people (aged 63-89) for user requirements gathering; 6 older people in field test (56-93 years old), 4 women 2 men  Selection from local clubs  Medical and care experts were also involved for requirements gathering  6 older people (aged 56-93) at laboratory test  Selection based on being cognitively intact, technologically literate, and suffering from a chronic but not debilitating disease | Focus groups, Interviews, think aloud protocols  Laboratory test  Field trial  Usability evaluation / prototype test | Role: experimental subjects, informants, testers  Level: low  Stage: early requirement gathering, iterative stages for testing and evaluation of prototypes  Image: Age-related illnesses, declining capabilities, patients, vulnerable | 7 needs of older users were identified during the focus group, including, amongst others, the ability to talk to families and medical caregivers, submitting alerts, and getting medication reminders (p.197)  According to the researchers, the smart home robot system was designed to address all identified user needs (p.201)  Usability evaluations and laboratory tests showed that the system and robot were considered likeable (p.199) and possibly helpful (p.200)  During the usability evaluation, the older people in the evaluation wished for the ability to decide on and manage the actions the robot could take (p.200f). In the following technical description of the system, such features are not included – instead, the robot was designed to be able to autonomously control the environment (e.g. temperature and light) (p.202) |
| Joshi and Bratteteig (2016) | Allow older people to influence design (p.4,7)  Design alternative technologies and improvements together with older residents (p.8)  Design technical support for old people to continue living independently in their homes (p.16) | 4 different supporting technologies: radio, information device, Kinect solution for physical exercise, indoor navigation system  Development from scratch and improve existing technology | A total of 70 people between 74 and 101 years old (average age 82)  Selection from long-term collaboration with municipality, as part of project “Care+”, a residence facility built for older people living independently in their own home, with installed welfare technology and some care workers for assistance. Recruitment based on snowballing: after first contact, participants were asked to find further interested people. Also, ad-hoc recruitment at reception area.  Daytime employees were also part of the workshops, as were representatives of municipalities | Interviews, observations, workshops, focus groups, usability testing, home visits, expert walkthroughs, exhibition, coffee meetings, rapid prototyping | Role: equal partner, consultant, informant, tester, co-designer  Level: high  Stages: iteratively, throughout all stages and design activities  Image: limited capacities for participating, reduced motor or cognitive capabilities, lack of technical expertise, but also competencies on their own lives | Different people had the choice themselves when to participate based on their abilities and interests (p.11f).  The participants differed in terms of the duration and frequency they joined, the breaks needed and their motivations to contribute (p.15)  The PD process helped the participants to learn about technological opportunities (p.11)  Two participants specifically valued the sense of ownership (p.14f), and many valued the direct sense of participation (p.17)  The older participants contributed by suggesting new technological opportunities, both independently and based on their experiences (p.17), by selecting among different alternatives (p.18), by concretizing (p.19) and evaluating choices (p.20).  The researchers say they succeeded in having the voice and say of the older people implemented in several design decisions. For example, the interface and knob of the DAB radio were adjusted to the older people’s preferences of a familiar interface and gesture of rotary controls for operating a radio. (p.21) |
| Kiat and Chen (2015) | Gather user needs (p.31)  Learn about user’s opinions and attitudes (p.31)  Develop a mobile application to make it more usable and accessible (p.29)  Gather feedback to improve the prototypes (p.36) | Mobile messaging application  Development from scratch, improvement of existing technology | Early user test with 2 older people (51 and 56 years old)  Focus group and user test with 6 older people (60-80 years), 5 of which were retired, 2 women 4 men.  Selection not further specified | Focus group interviews, Semi-structured interviews,  User testing  Prototype test and evaluation | Role: informant, tester, experimental subjects  Level: low  Stage: early requirements gathering, iterative prototype tests  Image: at risk of loneliness, limited abilities | Based on the input by the older people, several user requirements were specified, such as a bigger font size and less confusing icons (p.32)  The gathered requirements were implemented in the design, for example a clearer redesign of the icons (p.33) and the ability to choose the preferred font size (p.32).  During the first evaluation, those adjustments were appreciated by one of the participants (p.33). He also gave some additional feedback that was implemented in the second version of the prototype. (p.33)  In the third iteration, another participant gave feedback that another button was confusing, which was subsequently changed accordingly in the fourth iteration. The participant otherwise liked the interface changes (p.34f)  In a fourth iteration, another participant found the prototype easy to use and considered recommending it to others (p.35). |
| Kopeć et al. (2018) | Let older people participate in process (p.1895)  See if their participation affects the development process and the quality of the product (p.1896,1906), dispels stereotypes (p.1897), and how it affects the social dynamics (p.1896).  Empower older users (p.1900)  Learn about their personal insights and experiences (p.1904), their needs and visions (p. 1915) | Mobile applications (various, schedule intake of drugs, social activity plasnning, volunteer service)  Development from scratch | 15 older people, 11 women 4 men, mean age 68 years, mostly retired, college degree, experienced with technology  Selection from connection with LivingLab at Academy of Information technology from university  81 younger participants, computer science students, also participated | Joint Hackathon, which the researchers observed, complemented by focus group interviews, in-depth interviews, and surveys  The final designs were evaluated by a jury.  The jury consisted of 15 representatives from diverse backgrounds: academics NGO, IT professionals and business.  The final rank was the combination of all of the jury members’ individual rankings. | Role: none, consultant, co-designer, equal partner  Level: **all three, depending on team**  Stage: idea generation & app development, user requirement and evaluation of progress  Image: It is a stereotype that older people are not skilled with ICT. Some young people believed that older people are in general not experienced with technology, as they claimed the older people that were included were too experienced to be representative of all old people.  Older people also applied stereotypes to themselves. | There were three types of collaboration: isolation, consultation and full collaboration (p.1919). Two teams that fully collaborated older adults won (first and second place), but three teams that fully cooperated with older adults did not win (p.1907, 1910).  The findings suggest a positive effect of the collaboration scenario on the software quality. The hackathon teams that used the “Full Cooperation” scenario were the most successful (p.1920) far ahead of the other ones (p.1905).  In the other groups, programmers did not treat the insights from older adults with full seriousness, because they thought their experiences were not representative of the whole population of aging people (p.1910), resorting to isolating older adults or only sporadically consulting them. Here, they resorted to stereotypes as a justification (p.1919)  Direct interaction reduced negative stereotypes and improved positive intergenerational attitudes (p.1919). The perceptions of both juniors by older adults and older people by juniors improved on nearly all aspects after the PD activities (p.1911).For the perceptions to improve, it made no difference if juniors merely consulted the older adults, or included them at their workplace (p.1912)  Winning teams frequently highlighted the importance of the older adults’ insights in developing their software. This was predominant in the teams that cooperated with their senior members as equal partners (p.1914) Older adults from the winning teams highlighted the importance of overcoming stereotypes for the development of a good software (p.1915).  Only two teams changed their initial plans to include the older people’s ideas and suggestions. These two teams were those who later won the competition (p.1909)  At least one of the winning applications is expected to be implemented in the community. (p.1922) |
| Le et al. (2014) | Learn about user needs (p.659)  Obtain feedback to increase perceived value of smart home (p.658)  Develop visualization in line with older people‘s needs (p.658) | Visualization of smart home sensor system  Development from scratch | 8 community-dwelling older adults (at least 65 years old)  Selection not further specified | Interviews | Role: informant  Level: low  Stage: requirements gathering  Image: risk of health problems, age-related decreasing cognition and mobility | Based on the replies of the older users, the designers focused on developing longitudinal visualizations of sensor data and higher granularity of information, because older people were interested specifically in long-term trends and patterns they were unaware of (p.662). |
| Lee et al. (2017) | Facilitate mutual learning (p.244)  Let older adults actively participate (p.244)  Design robot to be successfully used in social environment (p.244)  Increase acceptance of robot (p.244)  Empower older adults (p.246, 250)  Provide older adults with information (p.248)  Learn about how older adults perceived different robots (p.248)  Develop concepts and scenarios how robots can be used in their home (p.248)  Develop own robot designs based on their daily experiences (p.249) | Socially assistive robots  Improve existing robots | 5 older adults (58-71 years old), living at home, rural and of lower socio-economic status, 2 women 3 men, experienced with computers and 1 still working  Selected from a local outpatient healthcare provider  10 clinical and caregiving staff also participated | Semi-structured interviews, 4 participatory design  Workshops / Focus groups | Role: informants, co-designers  Level: intermediate  Stage: requirements gathering, concept and idea generation, technical development  Image: diagnosed with depression and physical illness, patient, not technically savvy; but experts on local conditions and needs | Mutual learning occurred as older adults learned about existing robots, and designers learned about the difficulties of older adults experiencing aging and depression (p.247)  The designers learned about how older adults perceived robots (p.248), as well as how older adults related the robots to their personal needs (p.249). For example, older people did not want the robot to go into private places and be able to maintain control over the robot (p.249), and did not want to share private information with medical professionals (p.250).  The older adults made several suggestions for potential uses of a robot, like providing reminders and suggesting healthy nutrition (p.249), or provide companionship by being able to discuss the news or talk to the robot (p.248).  According to the authors, the older adults also learned to envision robots in comprehensive ways as a whole set of interacting functions in the use context (p.249). They claim that in the end, participants more often expressed their suggestions from a design point of view (p.250).  Participants enjoyed the workshops as places where they could socialize and where their voices were heard (p.251). |
| Leong and Johnston (2017) | Design technologies that suit older people’s needs (p.702f)  Empower older people (p.702)  Ensure mutual learning (p.703)  Learn about user’s needs (p.706)  Elicit older people’s opinions and experiences (p.708) | Companion robot  Development from scratch | For first 3 workshops & interviews: 8 healthy older people (65-75 years), 4 women 4 men.  Further 2 workshops for evaluation: 16 older people (65-90 years old)  Selection not further specified. | Face-to-face Interviews, 5 PD workshops  Prototyping | Role: informants, consultants, co-designers  Level: intermediate  Stage: early stage, idea generation, evaluation  Image: avoid skewed stereotypes of aging, instead: healthy, active, and independent | The idea of a robot was not in the designers’ minds before engaging the older people (p.703)  Through the interviews, the designers learned about the older people’s live experiences, hopes and dreams (p.704).  The designers took the first workshop discussion about the benefits of dogs as companions as an inspiration for exploring the idea of a robot dog (p.705)  According to the researchers, the older people were able to envision and articulate possible functionalities they will use, that are suitable and meaningful to their needs and aspirations (p.709)  Based on the second workshop, the designers captured the old people’s ideas for possibly useful functions and scenarios (e.g. to have the robot as a companion, and connected with mobile phones) and developed scenarios (p.707)  As a result of the third workshop, feedback was obtained from the older adults how they wish to interact with the robot dog, for example by having consistent responses, and customized control over the robot dog (p.708)  In the following workshop, the developed design idea and scenario of the robot dog was evaluated by other older people, who mostly liked the robot (14/16), however imagined its possible use not to replace real dogs, but in homes were real dogs were not allowed. (p.709)  The participants did not consider it problematic to interact or live with a robotic dog (p.710) |
| Maaß and Buchmüller (2018) | Increase acceptance (p.119)  To learn about user needs (p.121)  Understand older people’s experiences during retirement (p.121)  Design software system that meets older people’s expectations and requirements (p.119) | Online neighbourhood platform, software application  Development from scratch | 15 retired older adults (57-75 years old), 11 women 4 men.  Selection not further specified. | Cultural probes, Interviews, 4 workshops, prototype evaluation  Alternating between joint work with the participants and development by the project team | Role: informants, consultant, co-designer  Level: intermediate  Stage: requirements elicitation, idea generation and concretizing, prototype evaluation  Image: problems regarding social networks, physical impairments, however, also that older people are experts of their live situations | The involvement of the older people significantly altered the initial design focus. Originally, the designers had the idea of creating an intergenerational neighbourhood platform to enable young and old people exchange and support their ideas. Based on the reflections by the older adults and the subsequent discussions, a prototype of a digital neighbourhood system was developed, where older people can communicate with each other to share and appreciate their experiences of retirement (p.134).  Furthermore, the researchers gained insights into the participant’s daily lives (p.124). Those insights served as inspiration for personas and scenarios (p.126). Workshops helped to further elicit needs and ideas of the participants. Here, it became clear that the participants did not look for young persons, but rather liked to meet peers reflecting their own situation, to jointly learn, get inspired, share experiences and eventually meet in person (p.126). These ideas were then considered by the designers to alter their original design idea and develop several different paper prototypes (p.126).  The design workshops were dominated by the researchers through the pre-defined prototypes (p130f). However, the older people could evaluate, extend and refine those in a third workshop (p.128). Here, they contributed by emphasising the importance of being able to trust unknown people they meet online, including the requirement to identify themselves and provide basic information, as well as not revealing too much about themselves (p.129). The participants used these insights to decide how to design the profile on the platform.  Finally, a digital prototype was developed incorporating their previous ideas, focusing on meeting like-minded older people and being able to determine the visibility of their data (p.131).  The older adults were positive about the results of what has been incorporated, and expressed their increased interest in online platforms (p.131f)  The older adults appreciated their involvement in the design project, as they managed to learn more about themselves (p.125) and felt being treated as an equal (p.133).  However, the platform has not been implemented, which made the participants disappointed (p.132) |
| Mehrrotra et al. (2016) | Learn about older people’s practices and experiences (p.91)  Obtain feedback on different prototypes (p.91) | Social robotic interface, embodied conversational agent  Development from scratch | For survey, 22 older adults (mean age 67.43), 11 men 11 women, contacted over internet  For focus groups, 14 older adults (mean age 71.32), 9 men and 5 women  Selection not further specified. | Survey, focus groups, Interviews, rapid prototyping  Claimed to be PD but more UCD. | Role: informants  Level: low  Stage: requirements gathering  Image: burden on healthcare system, hearing and visual impairments | Based on the interviews and focus groups researchers learned about older people’s practices, i.e. some taking note of their routines, and preferences, i.e. them not wishing the robot to replace humans (p.92).  In the interview, most people preferred an animal-like robot (p.92), followed by a human-like robot. However, afterwards, the researchers designed only prototypes of a human-like and a plant-like robot (p.93). In the subsequent feedback session, the human-like robot gained the most positive feedback (p.93). |
| Müller et al. (2015) | Learn about older people’s lives and attitudes (p.2297)  Teach older people about use of technology and get over their scepticism (p.2299)  Adjust design to older people’s needs (p.2299)  Enable acceptance and adoption of neighbourhood platform (p.2299) | ICT-based neighbourhood portal  Use existing technology to develop new platform | At least 15 older people (60-86 years old), community dwelling  At least 5 younger people (30-55 years old), community dwelling  Other participants are a local housing company, a counselling agency and HCI university researchers.  Selection of older people from local housing complex. | Interviews, several iterations of workshops, prototype evaluation  Claims to be PD, but older people were mostly confronted with ready-made options to evaluate. | Role: informants, consultants  Level: intermediate  Stage: Context research, prototype evaluation and refinement  Image: fear of technology, technologically illiterate, non-tech-savvy, in need to be educated, reluctance, disinterest | In the interviews, the researchers found a “high reluctance” among older people towards technology (p.2298)  The designers implemented the feature “organizing common activities” based on the suggestions of the older people in the workshops to want to be able to offer help (p.2301)  The researchers also took seriously the worries of older people about issues of trust and privacy, and enabled a feature in the platform to select with whom to share information (p.2301).  Furthermore, young people suggested a feature to be able to rate people in the community. Here, the researchers considered the comment by the older people regarding the fear of loss of reputation, and enabled a feature in the platform to thank helpers, but not to discredit them (p.2301).  According to the researchers, the software is now used for evaluation purposes by the older tenants (p.2301).  According to them, the older people also enjoyed seeing how their ideas became implemented into the design (p.2302) |
| Nielsen et al. (2018) | Learn about user needs (p.405)  Build eHealth application in line with user needs (p.405)  To improve quality of future technology (p.405) | eHealth application to manage hearing impairment  Development from scratch | 36 older persons with hearing impairment (61-81 years old), 16 women 20 men  Selection online through questionnaires, purposive sampling based on that they use hearing aids for a given time frame and are at least 60 years old, with specific gender ratio  Significant others for the older persons and audiologists also participated in the first focus group | Interviews, Focus groups, prototype evaluation | Role: informant  Level: low  Stage: User requirements gathering, prototype evaluation  Image: not all older people are tech-savvy, hearing impairment, participants with valuable knowledge | The researchers learned that older people desired the ability to personalize information, learn about hearing-related aids, and being able to communicate with audiologist if necessary (p.407)  The participants rated different features to be important to them, and the designers focused on three of them, based on a thematic analysis of their replies. The first interactive digital prototype includes the older persons’ suggestions for being able to receive information in a personalized way, as well as for communication and learning (p.410).  Some features that older persons prioritized were, however, disregarded by the researchers, because they were already available, such as being able to fine-tune their hearing aids themselves (p.409)  In the final evaluation, some older people found certain features of the prototype confusing, while others requested additional features, for example the ability to perform self-reporting of tests and being able to see this over time (p.411f). |
| Pater et al. (2017) | Learn about facilitators and barriers to interventions meant to increase medication adherence (p.58)  Learn about user needs (p.64)  Create a technological solution that is based on older people’s perspective (p.64) | Medication reminder technology  Improvement of existing technology | 63 older people (56-85 years old) with hypertension, 41 women 22 men, of which 10 participated in the PD session  Selection from state-specific Tech HomeLab, part of network, based on that they had hypertension, have at least three prescribed medications, and managed their own medications | Interviews, questionnaires, observations,  User test,  Participatory design session | Role: informant, tester, co-designer  Level: low  Stage: User requirements gathering, idea generation, user test  Image: Older people with hypertension; health risk; declining cognitive abilities | The researchers found out about the impact of medication on older people’s life, the reasons why self-report was missed and different triggers that work for them (p.62)  The test showed the technology developed without user involvement caused no change in behaviour (p.63)  As a result of the PD session, the older people suggested a smart pill box combined with mobile notifications to be the most preferred way of receiving reminders, allowing the pill box to show lights and make sounds, and being able to pause reminders if they are too frequent (p.64f).  Additionally, a couple of other needs were identified, e.g. flexibility of alarm sounds or feedback reports over time (p.65) |
| Pollmann et al. (2018) | Learn about user needs (p.52,56, 57)  Develop product in line with older people’s needs (p.52,56)  Increase acceptance (p.56)  Evaluate and extend the idea of a virtual companion (p.61) | Virtual companion  Development from scratch | 63 older adults (60-95 years old), 25 women 38 men, of which 4 participated in the prototype evaluation, 2 women 2 men; majority retired but also some still working  Selection not further specified | Semi-structured interviews, survey, prototype evaluation, workshop  Highly structured, strong role of designers | Role: informant, tester  Level: low  Stage: Requirements gathering, prototype evaluation  Image: not ‘naturally’ inclined to accept technology | The replies by the older people in the interviews were analysed and matched with some pre-existing UX framework. Out of this, three needs were extracted: connectedness, stimulation and competence (p.58)  In the workshop, participants were guided to select needs out of this framework and build a prototype with Lego, and were asked to spend a week with that prototype and report their experiences (p.61f). Their feedback was quantitatively analysed, and most important needs were elicited, amongst others the ability to store memories and motivate physical activities, stimulation and self-expression (p.63).  According to the authors, this helped them refine the requirements they should focus on (p.64). |
| Raviselvam et al. (2016) | Learn about older people’s needs (p.132)  Develop a design based on their needs (p.132)  See if this design is universally appreciated (p.132) | Redesign of water bottle cap, sewing needle, mattress and soda cans  Improve existing technology | 34 older adults (aged at least 65 years) in need identification & design ideation phase  30 older adults in prototype evaluation phase  Selection of older adults from different local senior activity centres based on approval from the centre manager. Older adults were also recruited at other locations, e.g. outside shopping malls and in public spaces. | Interviews, Surveys, Focus-groups, prototype evaluation | Role: informant, consultant, tester  Level: low  Stage: need identification, ideation, prototype evaluation  Image: lead user experiencing needs more clearly, due to physical loss of aging | Based on the comments by the older people on how difficult they perceived different tasks, and perceptions of feasibility by the designers, four features were selected for improvement: water bottlecap, sewing needle, mattress and soda cans (p.134)  Products were redesigned based on suggestions and inputs by older people, e.g. soda cans with deeper dent and using different materials to fasten mattress (p.135f)  90% of older adults and 89% of the younger people preferred the redesigned products over existing ones. According to the authors, this shows that the needs experienced by older adults could, to some extent, indicate the latent needs of the general population, and that involving older people may result in innovative ideas that might be accepted by the general population as well. (p.139) |
| Righi et al. (2017) | Learn about older people’s everyday life and needs (p.18)  Explore design opportunities for games and social connection (p.18)  Encourage adoption (p.18) | 2 digital technologies: Social networking platform, &  Online gaming platform  Development from scratch | 390 older people (55-81 years old), of which 18 met weekly in courses meetings for network platform, and 100 met in 9 workshops for the development of the gaming platform  Selection through local grassroots organisation aimed at providing education to the people living nearby. The NGO helped in identifying and contacting interested participants. The researchers joined and extended ongoing courses, created new courses or ran activities in public events that the organisation organized to gain attention. | Participant observation, ethnography, diaries, focus groups, informal face-to-face interviews, ICT courses, PD workshops, weekly meetings, questionnaire | Role: informant, consultant, co-designer  Level: intermediate  Stages: contextual analysis, participatory design, prototype evaluation  Image: at the beginning of project: older people as large user group, generic, homogenous based on age, in need of help, need to stay socially connected, players  After project: raise awareness of positive image of old age, heterogeneous, socially active individuals, both players and creators | The ethnographic insights were used by the designers to develop the two design concepts (p.18)  Through the continued involvement of older people, the initial design images evolved and were reinterpreted significantly (p.20).  For example, the older people actively contributed in the co-design activities of the network platform to provide help, but help for others. Through continued involvement, the designers became aware that older people did not think of themselves as potential users of this technology. The technology only was used when ‘help’ was reframed as ‘knowledge exchange’ (p.21).  Similarly, the comments by the older people were considered that they did not want caregivers as target users, and that they did not want it to only be ‘for older people’, because they saw themselves as active and healthy people. They preferred the service to be for the entire neighbourhood, and this was also implemented by the designers (p.21).  The heterogeneity in playing interest also changed the focus of the game design, allowing for different older people to individually create their own game (p.21)  However, different older people showed different interests in using the platform, and used it in different ways – some accepted it while others did not (p.22), meaning just having it designed together was not a sufficient criterion for uptake. The people who were not interested often voiced being annoyed with the game. |
| Stein et al. (2017) | Understand factors that facilitate adoption (p.5716)  Learn about participant’s transportation habits (p.5719)  Identify opportunities for ICT-based support (p.5719)  Obtain feedback on prototype (p.5719) | Digital transportation information platform: mobile, web and iTV application  Design from scratch | 23 older adults (58-82 years old), 21 in context study and 19 in co-design/evaluation stage (2 dropped out, 2 were replaced), living at home  Selection from local city | Interviews, focus groups, Co-design workshops, prototype test and evaluation  Specify they conduct co-design workshops but do not describe this part of design. | Role: informants, testers, consultants  Level: low  Stage: context study, evaluation study  Image: diminished driving capabilities, reduced financial resources, shrinking social network | The authors, using content analysis of the interviews, learned about the older people’s transport choice behaviour (p.5720), for example a desire for operational knowledge and the ability to retrieve information based on situational circumstances (p.5721). The designers say that these considerations helped them when developing the first prototype for the workshops, e.g. a focus on the situational use (p.5724)  Generally, the prototype was positively evaluated. Older participants used the device mainly for planning in advance. They appreciated it, for example because it reduced uncertainty and facilitated access to more information (p.5722f).  Factors relevant for adoption were: reducing uncertainty, complementing transport information with context information (e.g. event calendar) and providing informational access based on the situational needs (p.5725) |
| Teixeira et al. (2017) | Obtain feedback on prototype (p.547)  Develop application that meets the needs and features of older users (p.547) | Mobile application to increase medication adherence among older adults  Development from scratch | 4 older people (57-76 years old), 3 women 1 man for second round of evaluation, 10 older people (57-82), 9 women 1 man, for third round of evaluation; mostly retired and high education  Selection from senior university, according to consecutive recruitment  Engineers and health professionals evaluated in the first round | Survey, Prototype evaluation & test | Role: tester, experimental subject  Level: low  Stage: 2 rounds of evaluating  Image: high disability, physical and cognitive impairments, memory loss, inherent demotivation | Older people provided feedback and made suggestions for improvement, for example to add a tutorial and improve the voice interaction (p.554).  The prototype was adjusted based on these comments (e.g. choice of different voices). One major new feature was the option to add new drugs to the medication plan (p.555)  The final evaluation showed a high score on ease of use and consistency, while ease of learning had a low score (p.556). |
| Verhoeven et al. (2016) | Learn about older people’s everyday experiences and practices (p.33)  Design useful application (p.33)  Design application based on older people’s capabilities (p.33)  Gather information about requirements (p.34)  Gather feedback (p.35) | Mobile application to stimulate mobility among older people  Development from scratch | In first stage: 21 Dutch older people (58-93) living independently, 13 Spanish older people at least 65 years old  In evaluation stage: 12 older Dutch people (Mean: 68.5 years), 8 women 4 men  In mock-up test phase: 9 older Dutch people (60-70 years old), 4 older Spanish people (60-80 years old)  Selection not further specified (apart from age) | ‘PD sessions’ (for req gathering), cultural probes, Interviews, Questionnaire,prototype evaluation, mock-up test | Role: informant, tester  Level: low  Stage: learn about user, prototype evaluation, mock-up test  Image: decreasing mobility, physical and cognitive loss, anxious and vulnerable, sceptical towards technology | According to the authors, the first phase revealed rich insights into older people’s lives, and they also made suggestions for service ideas. Based on these insights, scenarios and personas were developed, as well as prototypes (p.36f).  The older people also gave crucial comments in the evaluation, e.g. that they would not want to carry a mobile phone or tablet (p.40), not too many reminders (p.41) and a bigger font size (p.42). |
| Wallisch et al. (2018) | Learn about user needs, attitudes and behaviours (p.1396-1398)  Design technology in line with user needs (p.1393, 1398) and everyday practices of older people (p.1395) | Transportation solution: Tricycle kit  Development from scratch | 10 older people (68-88 years old), 4 women 6 men; day care residents  Selection from local day-care centre | Focus group  It was specified at the onset that they wanted to develop a solution similar to a common bicycle to maintain a healthy lifestyle of older people travelling short distances. | Role: informant  Level: low  Stage: intermediate requirements gathering  Image: fear of uncertainty, routine-based, sceptical to change, have special needs, heterogeneous | The comments by the older people helped to identify some requirements, e.g. affordable price and familiarity (p.1400)  The designers considered the older people’s diverse wishes, for example differently shaped handlebars, and combined it with the common desire to keep the bicycle that is familiar for them. Hence, they designed a tricycle kit that can be adapted based on the older people’s preferences to the bicycle they already own (p.1400)  In a following evaluation, the older people indicated a higher intention to use this kit than other bicycle versions (p.1401), though actual adoption has not been evaluated. |
| Willard et al. (2018) | Assess needs of older people who are frail (p.2)  Develop a platform in line with older people’s needs (p.7)  Test the platform to learn about user experiences, usability and feasibility (p.4,7) | Online community care platform  Improve / adjust existing technology for older users | 17 older adults at least 65 years old during requirement gathering, 11 women 6 men  Selection of these 17 adults through different organisations (e.g. a client-interest group, a hospital, and a local municipality)  73 older adults at least 65 years old during prototype test but of which only 55 were included  Selection of these 73 people by means of an information letter distributed by senior council workers, a hospital, care organisations, welfare organisations, and general practitioners. Criteria were that they were older than 65, living independently, having some health problems or risk | Observation, Interviews, prototype test using log files and post-test interviews | Role: Informant, tester, experimental subject  Level: low  Stage: user requirement gathering, prototype test  Image: frail, at risk of further physical decline | The interviews and observations resulted in a couple of use cases and user requirements, e.g. having visible buttons and a feature for calling to enable social contact (p.5). These insights have been iteratively translated by researchers and ICT companies into a prototype (p.3).  Of the 73 test participants, 33 completed the entire monitoring period of 6 months. The frequency of use of the platform decreased over time (p.5). A majority of 73% of the older people did *not* consider the platform to have any added value (p.6) The authors speculate this might be because the participants saw themselves as being vital and active and not in need of the platform (p.6). However, this did not change their image, as they in the end suggested technical features of the platform to be further improved (p.8) |
| Wu and Munteanu (2018) | Enable acceptance (p.3)  Improve fall risk awareness (p.3)  Gather requirements for design (p.4)  Let older people share their ideas and perspectives (p.4)  Build technology based on older people’s input (p.4)  Improve the quality of the interface (p.4)  Gather feedback (p.6) | Mobile application displaying visualizations of sensor data and algorithmic assessments of fall risks  Development from scratch | 5 older people (57-67 years old), 3 women 2 men for PD sessions  4 older people (64-74) for field evaluation, 2 women 2 men  Selection through flyers distributed on the university campus, local libraries, and community centres. Criterion: comfortable using mobile touchscreens. | One-on-one participatory design sessions, prototype test& evaluation, interviews, surveys | Role: informants, consultants, testers, experimental subjects  Level: low  Stage: Requirement gathering, prototype test & evaluation  Image: at risk of falls but otherwise physically healthy | The participants made suggestions for features that the fall risk display should have, e.g. a long-term graphical display and instructions for preventing falls (p.5)  These suggestions, in total 9 out of 15, were implemented in the first prototype (p.5)  The evaluation shows that the mobile application had a positive effect on the participants, as it improved fall awareness, tendency to adapt fall prevention practices, and awareness of personal physical ability (p.7).  According to the authors, the technology is well accepted and adopted after being introduced to the participants, whose assessment is based on TAM indicators, perceived ease of use and perceived usefulness. 3 out of 4 testers would want to continue using it. (p.8-10) |

**PRISMA Checklist**

| **Section/topic** | **#** | **Checklist item** | **Reported on page #** |  |
| --- | --- | --- | --- | --- |
| **TITLE** | | |  |  |
| Title | 1 | Identify the report as a systematic review, meta-analysis, or both. | 1 (Title page) |  |
| **ABSTRACT** | | |  |  |
| Structured summary | 2 | Provide a structured summary including, as applicable: background; objectives; data sources; study eligibility criteria, participants, and interventions; study appraisal and synthesis methods; results; limitations; conclusions and implications of key findings; systematic review registration number. | 2 |  |
| **INTRODUCTION** | | |  |  |
| Rationale | 3 | Describe the rationale for the review in the context of what is already known. | 3 |  |
| Objectives | 4 | Provide an explicit statement of questions being addressed with reference to participants, interventions, comparisons, outcomes, and study design (PICOS). | 3 |  |
| **METHODS** | | |  |  |
| Protocol and registration | 5 | Indicate if a review protocol exists, if and where it can be accessed (e.g., Web address), and, if available, provide registration information including registration number. | A priori protocol is as approach described in paper (4-6) |  |
| Eligibility criteria | 6 | Specify study characteristics (e.g., PICOS, length of follow-up) and report characteristics (e.g., years considered, language, publication status) used as criteria for eligibility, giving rationale. | 5 |  |
| Information sources | 7 | Describe all information sources (e.g., databases with dates of coverage, contact with study authors to identify additional studies) in the search and date last searched. | 5 |  |
| Search | 8 | Present full electronic search strategy for at least one database, including any limits used, such that it could be repeated. | 4-5; Appendix A and B |  |
| Study selection | 9 | State the process for selecting studies (i.e., screening, eligibility, included in systematic review, and, if applicable, included in the meta-analysis). | 5 |  |
| Data collection process | 10 | Describe method of data extraction from reports (e.g., piloted forms, independently, in duplicate) and any processes for obtaining and confirming data from investigators. | 5 |  |
| Data items | 11 | List and define all variables for which data were sought (e.g., PICOS, funding sources) and any assumptions and simplifications made. | 5 |  |
| Risk of bias in individual studies | 12 | Describe methods used for assessing risk of bias of individual studies (including specification of whether this was done at the study or outcome level), and how this information is to be used in any data synthesis. | Qualitative analysis, pp. 5-6 |  |
| Summary measures | 13 | State the principal summary measures (e.g., risk ratio, difference in means). | Qualitative analysis, pp. 5-6 |  |
| Synthesis of results | 14 | Describe the methods of handling data and combining results of studies, if done, including measures of consistency (e.g., I^2^) for each meta-analysis. | Qualitative analysis, pp. 5-6 |  |
| Risk of bias across studies | 15 | Specify any assessment of risk of bias that may affect the cumulative evidence (e.g., publication bias, selective reporting within studies). | 5-7; 13 |  |
| Additional analyses | 16 | Describe methods of additional analyses (e.g., sensitivity or subgroup analyses, meta-regression), if done, indicating which were pre-specified. | No additional analyses |  |
| **RESULTS** | | | |  |
| Study selection | | 17 | Give numbers of studies screened, assessed for eligibility, and included in the review, with reasons for exclusions at each stage, ideally with a flow diagram. | 6, Figure 1 |
| Study characteristics | | 18 | For each study, present characteristics for which data were extracted (e.g., study size, PICOS, follow-up period) and provide the citations. | 6 and table in Appendix C |
| Risk of bias within studies | | 19 | Present data on risk of bias of each study and, if available, any outcome level assessment (see item 12). | 6-7 |
| Results of individual studies | | 20 | For all outcomes considered (benefits or harms), present, for each study: (a) simple summary data for each intervention group (b) effect estimates and confidence intervals, ideally with a forest plot. | 7-11 and table in Appendix C |
| Synthesis of results | | 21 | Present results of each meta-analysis done, including confidence intervals and measures of consistency. | 7-11 and table in Appendix C; qualitative analysis |
| Risk of bias across studies | | 22 | Present results of any assessment of risk of bias across studies (see Item 15). | Qualitative, 6-7; 13 |
| Additional analysis | | 23 | Give results of additional analyses, if done (e.g., sensitivity or subgroup analyses, meta-regression [see Item 16]). | No additional analyses |
| **DISCUSSION** | | | |  |
| Summary of evidence | | 24 | Summarize the main findings including the strength of evidence for each main outcome; consider their relevance to key groups (e.g., healthcare providers, users, and policy makers). | 11-12, Figure 2 |
| Limitations | | 25 | Discuss limitations at study and outcome level (e.g., risk of bias), and at review-level (e.g., incomplete retrieval of identified research, reporting bias). | 13 |
| Conclusions | | 26 | Provide a general interpretation of the results in the context of other evidence, and implications for future research. | 12-13 |
| **FUNDING** | | | |  |
| Funding | | 27 | Describe sources of funding for the systematic review and other support (e.g., supply of data); role of funders for the systematic review. | 2 |

*Adapted From:*  Moher D, Liberati A, Tetzlaff J, Altman DG, The PRISMA Group (2009). Preferred Reporting Items for Systematic Reviews and Meta-Analyses: The PRISMA Statement. PLoS Med 6(6): e1000097. doi:10.1371/journal.pmed1000097
